# Precision Medicine in Osteosarcoma: MATCH Trial and Beyond

**DOI:** 10.3390/cells10020281

**Published:** 2021-01-31

**Authors:** Elisa Tirtei, Anna Campello, Sebastian D. Asaftei, Katia Mareschi, Matteo Cereda, Franca Fagioli

**Affiliations:** 1 Paediatric Onco-Haematology Division, Regina Margherita Children’s Hospital, City of Health and Science of Turin, 10126 Turin, Italy; campello.anna@gmail.com (A.C.); sebastiandorin.asaftei@unito.it (S.D.A.); katia.mareschi@unito.it (K.M.); franca.fagioli@unito.it (F.F.); 2 Department of Public Health and Paediatrics, The University of Turin, Piazza Polonia 94, 10126 Turin, Italy; 3 Cancer Genomics and Bioinformatics Unit, IIGM—Italian Institute for Genomic Medicine, c/o IRCCS, Str. Prov.le 142, km 3.95, 10060 Candiolo (TO), Italy; matteo.cereda@iigm.it; 4 Candiolo Cancer Institute, FPO—IRCCS, Str. Prov.le 142, km 3.95, 10060 Candiolo (TO), Italy

**Keywords:** osteosarcoma, precision medicine, next generation sequencing, target-therapy, clinical trials

## Abstract

Osteosarcoma (OS) is a rare bone malignant tumour with a poor prognosis in the case of recurrence. So far, there is no agreement on the best systemic therapy for relapsed OS. The availability of next generation sequencing techniques has recently revolutionized clinical research. The sequencing of the tumour and its matched normal counterpart has the potential to reveal a wide landscape of genetic alterations with significant implications for clinical practice. The knowledge that the genomic profile of a patient’s tumour can be precisely mapped and matched to a targeted therapy in real time has improved the development of precision medicine trials (PMTs). PMTs aiming at determining the effectiveness of targeted therapies could be advantageous for patients with a tumour refractory to standard therapies. Development of PMTs for relapsed OS is largely encouraging and is in its initial phase. Assessing OS features, such as its rarity, its age distribution, the technical issues related to the bone tissue origin, and its complex genomic landscape, represents a real challenge for PMTs development. In this light, a multidisciplinary approach is required to fully exploit the potential of precision medicine for OS patients.

## 1. Introduction

Osteosarcoma (OS) is a rare mesenchymal tumour [1] and the most common primary malignant bone tumour in adolescents and young adults [2]. Despite the innovations in molecular medicine in recent decades, there has been little progress in the treatment of OS for over 30 years [3]. When the disease is localized, the surgical resection of the primary tumour and multiagent chemotherapy (e.g., high dose methotrexate, adriamycin, and cisplatin with or without ifosfamide) allows for a cure rate of 60–70% [1,2,4]. When the disease presents metastases by the time of diagnosis or in the case of relapsed disease, the prognosis for the patient is poor, and the survival rate is lower than 30% at 5 years [1,2,4,5].

Despite the consensus on the first line treatment of patients with systemic regimens, when recurrence or progression occurs, there is no international agreement on the best therapy for OS patients [6]. With the exception of the benefits of a surgical complete remission (second or subsequent) [4,5,6], there continues to be an ongoing debate regarding the efficacy of systemic treatments [4,5,6] and there is a lack of efficient treatment options for patients with advanced and relapsed OS.

The availability of Next Generation Sequencing (NGS) techniques has completely revolutionized clinical research, as well as basic and applied medicine over the last decade [7]. NGS is a high-throughput sequencing technology that is able to precisely map the entire genome of an individual in a few hours with a limited cost [8]. NGS approaches include whole genome sequencing, exome sequencing, transcriptome sequencing, and RNA sequencing [8,9]. These methodologies have facilitated the analysis of genomic profiles in cancer patients improving our understanding of the disease [7,9]. The tumour sequencing and its matched normal counterpart has the potential to reveal a range of genetic alterations with significant implications for clinical practice in terms of diagnosis, prognosis and subsequent treatment choices [10]. Indeed, NGS allows the identification of tumour specific aberrations with the potential either to discover new prognostic biomarkers or to offer potential information for new personalized treatments, thus improving the precision medicine concept especially for patients with a relapsed/refractory disease [7,10].

The development of precision medicine trials (PMTs) has improved as a result of the knowledge that the genomic profile of a patient’s tumour can be precisely mapped and matched, when possible, to a targeted therapy in real time [3]. Initially, PMTs were most common for adult malignancies, especially for the most frequent neoplasms. Thereafter, there was a gradual interest in expanding this personalized approach to rare tumours such as paediatric tumours, including OS [3,11].

So far, standard clinical trial approaches for relapsed or advanced OS patients showed a lack of efficacy [1,2,6]. The identification of specific genomic targets for OS patients is essential for better stratification of the patients enrolled in future matched clinical trials [12].

It is now widely accepted that OS patients may benefit from a deep comprehensive molecular genomic sequencing approach [3,13]. Nevertheless, this kind of treatment approach is in its infancy, and there are several obstacles to overcome for OS patients as described below.

## 2. Oncological Precision Medicine Trials

Several oncology studies have evaluated the feasibility and use of genomic-driven precision medicine in tumours in recent years.

Molecular Analysis for Therapy CHoice (MATCH) is a precision medicine treatment clinical trial for adult and paediatric patients supported by the National Cancer Institute (NCI) and by the Children’s Oncology Group (COG). The trial has an umbrella design with treatment rules based on the presence of a molecular aberration [11,14,15]. This study seeks to determine the response rate in adult and paediatric patients with advanced and relapsed solid tumours harbouring genomic alterations that can be treated with available drugs [11]. Following the same principles, further PMTs have been established along the same lines, including some for paediatric patients (Table 1).

PMTs usually consist of three phases. The first phase includes sample collection (especially tumour biopsies), the preparation of biological materials, and the generation of all required genomic data. As a result of these, each tumour of all enrolled patients is univocally associated with a precise molecular profile. During the second phase, genomic data is assessed by an expert multidisciplinary board to evaluate their clinical relevance. This panel of experienced clinicians evaluates the genomic alterations of each tumour considering their biological relevance, their potential therapeutic targeting with available compounds, and their link with the medical history of the patient. These evaluations are necessary for a rapid translation into a clinical decision making. Therefore, whenever possible, the enrolment of the patient into a genomic-driven treatment trial is encouraged.

The second phase aims at identifying the genomic alterations that are relevant for tumour growth and progression that could be selected for a targeted therapy. This clinical research employs computational algorithms able to prioritize the potentially druggable alterations identified from NGS data. In this light, Worst and colleagues developed a work-flow able to rank genomic alterations in seven levels of increasing clinical relevance (e.g., from “very high” to “very low”) on the basis of their effect on the encoded protein, the availability of a direct targeting drug, and literature evidences of possible pathway activation [17]. In the INFORM pilot study, patients with a very high priority target alterations (e.g., ALK, BRAF, and NRAS mutations, and MET and NTRK-fusions) showed an improvement in the Progression-Free Survival (PFS) [18]. Indeed, the median PFS in paediatric patients with a high priority genomic alteration treated with the matched drug was significantly higher compared to that of all patients with no druggable aberration (i.e., 204.5 and 114 days, respectively—*p* = 0.0095) [18]. Hence, a promptly identification of patients whose tumours are harbouring a high priority target genomic alteration is necessary to better address the physician’s treatment choice.

The preliminary results of the genomic-driven precision medicine trials, despite being promising, also presented us with a two-sided challenge. Firstly, the results showed that feasible access to a genomic program to identify somatic targeted alterations is necessary. This can be achieved with a specialized team to translate the obtained genomic results into reasonable clinical action for those patients [8]. Moreover, the availability of molecular targeted drugs is necessary, especially for more vulnerable populations such as paediatric patients or patients with a rare oncological disease, including OS patients. Recent advances in our understanding of molecular oncogenesis allowed for the stratification of malignancies into molecularly similar tumours, both within and across the tissue of origin, thus leading to the establishment of improved therapeutic treatments [27]. This strongly suggests that the development of targeted therapies can be better informed by tissue agnostic clinical trials, which represent a significant paradigm shift in precision medicine for cancer patients [27].

## 3. Osteosarcoma: The Challenges for Successful PMTs

OS patients represent a minority of patients that have been enrolled into PMTs so far (Table 1). This low enrolment rate is due to some challenges that prevent us from fully exploiting the beneficial impact of PMTs on OS patient quality of life. In the following paragraphs, we will discuss three important hurdles for a successful OS PMTs: (1) The modest incidence rate of this rare tumour [9], (2) the technical issues to manage genomic materials from a difficult tissue of origin (i.e., bone) [8], and (3) the highly heterogenous genomic complexity of OS samples [8,28,29,30].

### 3.1. Osteosarcoma: A Rare Tumour

OS is a rare tumor and commonly occurs in adolescents and young adults [9]. The genomic landscape of paediatric OS is not distinguishable from genomic features in adult OS. So far, no differences have been found between childhood or adult OS in the frequency of potentially actionable alterations across samples, with respect to altered genes or distinct molecular subsets [8,31]. This could strengthen the collaboration between adult and paediatric oncologists to design aligned targeted-therapy clinical trials and PMTs enlarging OS cohorts.

### 3.2. Osteosarcoma: The Technical Issue of Dealing with Bones

In the last years, the collection of fresh and/or snap-frozen bone sarcoma tissue has been increasingly encouraged in the last years to overcome artefacts from decalcification, and, simultaneously, to foster the genomic characterisation of these tumours in the context of research programs [6]. Nevertheless, the processing of bone sarcoma tissue could be more difficult than expected due to the paucity of material from bone tissue biopsies, thus impacting on the clinical practice management.

### 3.3. Osteosarcoma: An Heterogenous Genomic Landscape

OS has a completely different genomic landscape from other sarcomas that are often characterized by a specific driven aberration, and more broadly by other paediatric cancers [8,28,29]. High genomic instability is a hallmark of OS genomics and is especially represented by one subcategory of instability known as chromosomal instability (CIN) [9]. CIN is the elevated rate of loss or gain sections or entire chromosome resulting in complicated structural, numerical aberration, and wide variability among tumour cells [8,30,32]. Consequently, high levels of chromosome structural variations (SV), elevated somatic copy number alterations (SCNA), but also rearrangements resulting from chromothripsis, as well as the hypermutated chromosomal region known as kataegis, are characteristics of OS. All these genomic features result in significant intra- and inter-tumour heterogeneity for OS, with a few recurrent clinically actionable alterations. Moreover, sequencing studies showed that OS accumulate non-silent somatic mutations at a rate of ~1.2 mutations per mega base pairs [33]. All these features are partially in contrast with the genomic landscape of other paediatric cancers that usually present a lower mutational and structural alteration rate [28]. The genomic landscape of OS samples reveals that SCNA have an important role to drive progression of the disease [8,34,35]. Nevertheless, the high rate of SCNA and SV made it hard to discriminate driver from passenger alterations, and this could represent an additional obstacle for identifying new molecular targeted therapies for OS [8,36].

The OS heterogenous genomic landscape also includes the immune-genomic features. The promising results of immunotherapy, including immune checkpoint inhibitors, in various cancers, have prompted new clinical trials in sarcomas as well [37]. Nevertheless a promising objective response rate has not been reported for OS patients yet [37].

Recent analyses showed that, globally, OS has a median immune infiltrate level lower than other cancers, with concomitant low T-cell receptor clonalities, whereas revealing a high immune-genomic inter-tumour heterogeneity [38,39]. OS can virtually be divided into three subgroups based on the level of immune infiltrate and its activity (i.e., low, intermediate, or high) [39]. Even in tumours with high levels of immune infiltrate, an ineffective immune response may be due to the lack of neoantigens or to the presence of tumour-intrinsic adaptive immune resistance mechanisms that allow for immune evasion or lack of T-cell activation only [39]. However, the identification of OS with high levels of immune infiltrates supports the rationale for developing biomarker-selected approaches to future immunotherapy trials in OS. In addition, it is necessary to explore new targeted therapies that can mitigate immunosuppressive mechanisms [39]. This concept highlights and supports the role of a precision medicine approach for immunotherapy as well.

Up to now, very few case-reports of OS patients treated for druggable genomic alterations have been described (Table 2). Notably, Subbiah et al. reported two patients with relapsed and metastatic osteosarcoma which did not benefit from the targeted therapy of the selected genomic alterations [3]. Drug resistance mechanisms in refractory disease, intra-tumoral heterogeneity, and a possible different genomic profile either between the primary and metastatic sites and the same tumour in different relapses may be responsible for the failure of the targeted therapy. Moreover, most of the alterations identified in OS have at the moment an unpredictable pathogenetic role, thus available target therapies may not act on pathogenic driver aberrations.

## 4. Target-Specific Clinical Trials for OS Patients

The role of second, or further-line systemic, therapy for recurrent OS is currently not defined, and there is no international agreement for treatment [6]. To overcome this limitation, wherever possible, OS patient enrolment in prospective studies should be encouraged. The clinical trial landscape for relapsed or advanced OS patients offers a heterogeneous repertoire of treatments that are not uniquely correlated with genomic findings.

Supplementary Table S1 shows the list of clinical trials that are presently active and recruiting relapsed and refractory OS patients, for which the inclusion criteria are clearly defined. The list does not include observational studies or clinical trials without any systemic therapy administration (e.g., surgical studies and radiological studies). The majority are early phase studies: 16 of them (27%) are phase I trials, 13 (21%) are phase I/II trials, and 29 (49%) are Phase II trials. Forty (67%) studies are designed for young patients including children younger than 12 years. Forty-four of these studies use targeted therapies, and 40% of them required a specific molecular feature as inclusion criteria (Table 3).

The majority includes patients with relapsed or refractory solid tumours; fourteen (23%) trials are specifically designed for OS patients. Nine of them use targeted drugs without requiring a documented molecular alteration as inclusion criteria. Regarding the forty-four trials with targeted treatment, 90% of them have a monotherapy treatment. The most frequent monotherapy treatments are tyrosine-kinase inhibitors (TKIs, 12 trials, 27%), followed by monoclonal antibodies (8 trials, 18%). Four studies use an association treatment strategy with a monoclonal antibody (PD1 or PDL1 inhibitor) and a further target-drug (TKIs or PARP-inhibitor or immunosuppressive drug) (Table 3).

## 5. Conclusions

The translation of genomic findings into clinical oncology continues to grow rapidly, offering novel promising choices of therapy for children and adults with cancer. For those patients presenting druggable genomic alterations these targeted treatments could significantly improve their life span and quality [18].

Over the last decade, the simultaneous advancement of two phenomena has revolutionized the clinical management of patients: (1) The availability of NGS techniques for a rapid identification of genomic aberrations and (2) the development of new target drugs [8,11,42]. Therefore, PMTs using clinical molecular testing have become more common for adult malignancies, and more recently for paediatric neoplasms as well [11]. A major challenge presented by PMTs is that the treatment arm is tailored for a small subset of patients with a specific genomic profile, and it is expected to detect a precise feature in a histology agnostic cohort based on a genetic marker [11]. Even if the reported results of the precision medicine approach for oncological diseases are encouraging, there are still obstacles to overcame, especially for histological subtypes such as OS patients.

NGS has allowed us to understand that the heterogenous genomic landscape of OS is completely distinguishable from other sarcoma that are often characterized by a specific driven aberration [29]. Although various pan-cancer genomic trials have described the role of single-nucleotide variants and small focal copy-number alterations in OS biology [35], the widespread somatic copy number alterations (SCNA), chromothripsis, kataegis, and aneuploidy have been clearly described as features of OS, and their role in tumorigenesis remains largely unknown [35]. OS is a heterogenous disease with a high degree of variability in patients [3,9]. For this reason, a major challenge to previous targeted therapies for OS patients has been in the accurate identification of targets within the individual patient [9].

It is widely debated that a biopsy might not be representative of the entire tumour lesion, but multiple and repeated biopsies are sometimes unrealistic for bone sarcomas [8,35]. Tumour heterogeneity is a significant issue for cancer genomic research and precision medicine approach, and it is therefore necessary to develop new techniques for a more precise analysis (e.g., single-cell analysis) [35]. For instance, traditional transcriptomic analyses are performed on the entire cellular population leading to a difficult identification of the contribution of specific cellular subtypes on the disease progression, thus avoiding the full characterization of intratumoural heterogeneity of OS [43]. Conversely, single-cell RNA sequencing has recently demonstrated promising results in exploring the intra-tumour heterogeneity of various cancers [43]. The current genomic clinical trials could only reflect the average measurements of gene mutation and expression profiling across the tumour cells, omitting the cell type composition, dynamics, and characteristics in OS tumour samples [43]. New solution, such as single-cell approaches, can reveal the wide cellular atlas of malignant cells and tumour micro-environment cell components generally included in each tumour sample. This sequencing solution may optimize the therapeutic target selection and the overall precision medicine approach [9,43].

However, further preclinical and clinical studies are required to evaluate the therapeutic efficacy of these genome targeted pathways [9] and considering alternative treatment schedules using targeted therapies alongside standard treatments throughout all phases of the disease.

The current genomic analysis and PMTs could impact the clinical research of the next future. All data, including genomic findings, toxicity, and efficacy of novel drugs, have a potential implication for optimizing future treatment decisions. Given the rarity and the genomic heterogeneity of OS, a multidisciplinary collaboration is required to fully realize the potential of a precision medicine approach for OS patients. The development process of new drugs and a new therapeutical strategy for OS requires new ways of thinking, improving a rapid identification of patients with a high priority genomic target, considering the use of novel drugs also at an initial phase of a standard treatment, and improving the implementation of nimble statistical design for future clinical trials. Meanwhile, a wide clinical data sharing and harmonization is needed for a comprehensive new drugs long-term toxicity awareness. A novel drug development process must include a strict patient follow-up in order to identify all toxicities (e.g., cardiological, neurological, pneumological issues, fertility, …). This clinical monitoring is important to improve the patient quality of life, especially for children and adolescents who receive a novel drug during their growth development period of life.

## Figures and Tables

**Table 1:** Published data of recent genomic programs including patients with OS.

Trial Title	Inclusion Criteria	N° Patients Enrolled/Analysed	N° Enrolled Patients with OS	N° Patients with One or More Actionable Alteration	N° Patients Treated with a Matched Therapy Based on Genomic Data	Ref.
Peds-MiOncoSeq	R/R tumours or rare tumours age: ≤22 yrs	102/91	29 patients with sarcoma (unspecified)	42	15	[16]
INFORM	R/R solid tumours age: <40 yrs	57/52	4 OS	39 *	10	[17]
INFORM update 2020	R/R solid tumours age: <40 yrs	1300/525	Not reported	444 (120 pts with very high or high priority target)	149	[18]
MOSCATO-01	R/R solid tumours age: <25 yrs	73/69	4 OS	42	14	[19]
BASIC3	paediatric tumours age: ≤18 yrs	150/121	4 OS	33	Not reported	[10]
PIPSeq	high-risk tumours age: ≤26 yrs	107/101	6 OS + 5 other sarcoma not more defined	38	6	[20]
iCat	R/R high-risk non-CNS solid tumours age: ≤30 yrs	101/89	11 OS	31	3	[21]
ClinOmics Program	non CNS solid tumours age: ≤25 yrs	64/57	4 OS	30	Not reported **	[22]
MBB	High risk or R/R solid tumors age: ≤22 yrs	60/58	4 OS	23	6	[23]
TRICEPS	R/R or hard-to-treat tumours age: ≤22 yrs	84/62	7 OS	47	9	[24]
MD Anderson Program	R/R sarcoma age: 8–76 yrs	102/102	11 OS	95	14 (only 1 OS pt)	[25]
NCI-MATCH Trial	R/R solid tumour, R/R lymphoma, R/R myeloma age: ≥18 yrs	6391/5540	255 sarcomas not more defined	31	21	[26]

R/R: Relapsed or refractory; CNS: Central Nervous System; yrs: years; OS: Osteosarcoma; pt: Patient. * 26 patients have one or more alterations with intermediate or higher priority score; ** in the paper it is reported that 24 patients were considered to have a mutation that was targetable in a clinical trial (12% matched at level 1 of NCI-Match Criteria in which the gene variant is approved for selection of an approved drug).

**Table 2:** Case-reports reported in literature of OS patients treated with a matched targeted drug on the basis of their tumour molecular finding.

	Tumour Histotype	Age/Sex	Actionable Alteration Considered for Target-Therapy/Methods	Drugs Administered	Response	Ref.
Patient 1	Metastatic OS refractory to 3rd CT line	21 yrs/female	- PIK3CA V344G (NGS) - c-MET amplification (NGS and IHC)	Metformin + Rapamycine + Crizotinib	PD	[3]
Patient 2	Metastatic OS refractory to 4rd CT line	16 yrs/male	- PDGFRA amplification (NGS and IHC) - TP53 loss (NGS)	Sorafenib + Bevacizumab + Temsirolimus (Phase I clinical trial NCT01187199)	PD	[3]
Patient 3	Localized OS refractory to 2nd CT line	37 yrs/male	- Positive staining of RANKL (IHC)	Sunitinib + Denosumab	SD after 18 months of treatment	[40]
Patient 4	Localized OS relapsed after 1st CT line	50 yrs/male	- Strong positive staining for VEGFR-2 (IHC)	Apatinib	PR at 11 months of treatment	[41]

CT = chemotherapy; yrs = years; NGS = Next Generation Sequencing; IHC = Immunohistochemistry; PD = Progression Disease; PR = Partial response; SD = Stable Disease.

**Table 3:** Main characteristics of the active clinical trial recruiting patients with relapsed/refractory OS.

CHARACTERISTICS OF THE TRIALS (N° tot: 60 Clinical Trials Registered on clinicaltrials.gov)	N	%
INCLUSION CRITERIA		
TUMOUR HISTOLOGY		
Only Osteosarcoma	14	23%
Bone Sarcomas	3	5%
All type of sarcomas	6	10%
All solid tumour	37	62%
AGE OF ENROLLEMENT		
<12 years	40	67%
<25 years	60	100%
PHASE		
I	16	27%
II	29	48%
I/II	13	22%
IV	2	3%
RANDOMIZATION		
Yes	9	15%
No	51	85%
LINE OF THERAPY		
1st line	5	8%
2nd line and other	49	82%
Maintenance phase	3	5%
All lines of therapy	3	5%
TYPE OF INVESTIGATIONAL DRUG		
Target Therapy	44	73%
Non Target Therapy	16	27%
TYPE OF TARGET THERAPY		
Tyrosine kinase inhibitor	11 *	24%
Monoclonal antibody	8 *	18%
CAR-T	6	13%
CDK inhibitor	5	11%
mTOR inhibitor	3	7%
Combination of two target drugs	4	9%
Others	8	18%
TARGET THERAPY ASSOCIATED WITH CHEMOTHERAPY		
Yes	16	36%
No	24	54%
CAR-T conditioning regimen	4	9%
REQUESTED TARGET-ALTERATION FOR ENROLLMENT		
None	42	70%
IHC specific positive staining	4	7%
Specific genetic alteration	14	23%
AGE OF ENROLLEMENT		
<12 years	40	67%
<18 years	56	93%
>18 years	60	100%

* the study NCT04351308 tests the efficacy of Camrelizumbab versus Apatinib in a randomized fashion. IHC = immunohistochemistry.

## Supplementary Materials

The following are available online at https://www.mdpi.com/2073-4409/10/2/281/s1, Table S1: List of clinical trials that are presently active and recruiting relapsed/refractory OS patients.
