# Supplementary material for: Precision Medicine in Osteosarcoma: MATCH Trial and Beyond
Source: Cells. 2021 Jan 31;10(2):281. doi: 10.3390/cells10020281 (PMC7911557; doi:10.3390/cells10020281)
Supplement: Supplementary file 1 [file cells-10-00281-s001.pdf]

**Supplementary Table 1: list of clinical trials that are presently active and recruiting relapsed/refractory OS patients**

| ClinicalTrials.gov NCT Identifier | Tumor Histotype | Phase | Randomization | Line of therapy | Requested Target-Alteration for enrollment | Investigational Drug | Target drug | Association with Chemotherapy | Eligibility Age (years) |
|---|---|---|---|---|---|---|---|---|---|
| NCT01669369 | OS | IV | Yes | First line | None | Lithium Carbonate | No | Yes | 8 -70 |
| NCT02013336 | Solid Tumor | I | No | Second and other | None | Irinotecan Sucrosofate Liposome | No | Yes | 1 - 21 |
| NCT02173093 | Solid Tumor | I/II | No | Second and other | None | Anti-CD3 x hu3F8 bispecific antibody (GD2Bi)-armed activated T cells | Yes | No | 1 - 29 |
| NCT02389244 | Bone Sarcoma | II | Yes | Second and other | None | Regorafenib | Yes | No | >10 |
| NCT02406781 | Solid Tumor | II | No | Second and other | None | Pembrolizumab | Yes | Yes | >18 |
| NCT02502786 | OS | II | No | Second and other | None | Hu3F8 | Yes | No | 1 - 40 |
| NCT02517918 | Solid Tumor | I | No | Second and other | None | Sirolimus | Yes | Yes | >13 |
| NCT02536183 | Solid Tumor | I | No | Second and other | None | Lyso-thermosensitive liposomal doxorubicin | No | | <30 |
| NCT02574728 | Solid Tumor | II | No | Second and other | None | Sirolimus | Yes | Yes | 1 – 30 |
| NCT02644460 | Solid Tumor | I | No | Second and other | None | Abemaciclib | Yes | No | 2 – 25 |
| NCT02945800 | ALL Sarcoma | II | No | Second and other | None | Nab-Paclitaxel + Gemcitabine | No | / | 12 - 30 |
| NCT03063983 | OS | II | Yes | Maintenance Therapy | None | Metronomic MTX plus cyclophosphamide | No | Yes | <30 |

| | | | | | | | | | |
|---|---|---|---|---|---|---|---|---|---|
| NCT03093909 | Solid Tumor | I | No | Second and other | None | Inhalatory Gemcitabine | No | No | 12 - 50 |
| NCT03155620/NCT04284774(tipifarnib) | Solid Tumor | II | No | Second and other | HRAS gene alterations | Tipifarnib | Yes | No | 1 - 21 |
| NCT03190174 | Solid Tumor | I/II | No | Second and other | mTOR Activating Mutations | ABI-009 + nivolumab | Yes | No | >12 |
| NCT03210714 | Solid Tumor | II | No | Second and other | FGFR1, FGFR2, FGFR3, or FGFR4 gene mutation | Erdafitinib | Yes | No | >12 |
| NCT03213652 | Solid Tumor | II | No | Second and other | ALK or ROS1 gene alteration | Ensartinib | Yes | No | 1 - 21 |
| NCT03213678 | Solid Tumor | II | No | Second and other | TSC1, TSC2, or PI3K/mTOR gene mutation | LY3023414 | Yes | No | 1 - 21 |
| NCT03213704 | Solid Tumor | II | No | Second and other | NTRK1, NTRK2, or NTRK3 gene fusion | Larotrectinib | Yes | No | 1 - 21 |
| NCT03220035 | Solid Tumor | II | No | Second and other | BRAF V600 gene mutation | Vemurafenib | Yes | No | 1 - 21 |
| NCT03233204 | Solid Tumor | II | No | Second and other | ATM, BRCA1, BRCA2, RAD51C, RAD51D mutations | Olaparib | Yes | No | 1 - 21 |
| NCT03277924 | ALL Sarcoma | I/II | No | ALL* | None | Sunitinib+Nivolumab | Yes | No | 12 - 80 |
| NCT03425279 | Solid Tumor | I/II | No | Second and other | None | Conditionally active biologic anti-AXL antibody drug conjugate | Yes | Yes | Phase I: ≥ 18<br>Phase II: ≥ 12 |
| NCT03449108 | Solid Tumor | II | No | Second and other | None | Autologous tumor infiltrating lymphocytes LN-145 | No | Conditioning regimen | 16 - 70 |
| NCT03449901 | Solid Tumor | II | No | First line + Second Line | None | ADI-PEG 20 | No | Yes | >10 |
| NCT03458728 | Solid Tumor | I/II | No | Second and other | None | Copanlisib | Yes | No | 6 months – 21 years |

| | | | | | | | | | |
|---|---|---|---|---|---|---|---|---|---|
| NCT03462316 | ALL Sarcoma | I | No | Second and other | IHC positive staining for NY-ESO-1 | NY-ESO-1 CAR-T | Yes | Conditioning regimen | 14 - 70 |
| NCT03478462 | Solid Tumor | I | No | Second and other | None | CLR 131 | No | No | 2 – 21 |
| NCT03507491 | Solid Tumor | I | No | Second and other | None | Nab-Paclitaxel | No | Yes | 6 months – 30 years |
| NCT03525392 | Solid Tumor | I/II | No | Second and other | None | 177Lu-3BP-227 | No | No | >18 |
| NCT03526250 | Solid Tumor | II | No | Second and other | Alterations in cell cycle genes and positive Rb expression by IHC | Palbociclib | Yes | No | 1 - 21 |
| NCT03598595 | OS | I/II | No | Second and other | None | Hydroxychloroquine | No | Yes | ≥12 |
| NCT03618381 | Solid Tumor | I | No | Second and other | EGFR expression | EGFR 806 CAR-T | Yes | | < 26 |
| NCT03628209 | OS | I/II | No | Second and other | None | Nivolumab | Yes | Yes | < 39 |
| NCT03635632 | Solid Tumor | I | No | Second and other | positive GD2 expression by IHC | C7R-GD2 CAR-T | Yes | Conditioning regimen | 1 - 74 |
| NCT03643133 | OS | II | Yes | First line | None | Mifamurtide | No | Yes | < 50 |
| NCT03676985 | OS | I/II | No | Maintenance Therapy | None | ZKAB001 | Yes | Yes | 18 - 55 |
| NCT03678883 | Solid Tumor | I/II | No | Second and other | None | 9-ING-41 | Yes | Yes | >18 |
| NCT03698994 | Solid Tumor | II | No | Second and other | MAPK pathway mutations | Ulixertinib | Yes | No | 1 - 21 |
| NCT03709680 | Solid Tumor | I | No | Second and other | None | Palbociclib | Yes | Yes | ≥2 - <21 |

| | | | | | | | | | |
|---|---|---|---|---|---|---|---|---|---|
| NCT03718091 | Solid Tumor | II | No | Second and other | None | M6620 (VX-970) | Yes | No | ≥ 12 |
| NCT03742193 | OS | II | No | Second and other | None | Apatinib | Yes | Yes | 10 - 50 |
| NCT03860207 | Solid Tumor | I/II | No | Second and other | None | Humanized anti-GD2 x anti-CD3 bispecific antibody (hu3F8-BsAb) | Yes | No | ≥ 2 |
| NCT03900793 | OS | I | No | Second and other | None | Sunitinib | Yes | No | 10 - 40 |
| NCT03932071 | OS | IV | Yes | First line | None | Zoledronic Acid | No | Yes | all age |
| NCT04040205 | ALL Sarcoma | II | No | Second and other | Abnormalities in the Cyclin D1 - CDK4/6 - Rb pathway | Abemaciclib | Yes | No | ≥ 18 |
| NCT04055220 | Bone Sarcoma | II | Yes | Maintenance Therapy | None | Regorafenib | Yes | Yes | ≥ 16 |
| NCT04154189 | OS | II | Yes | Second and other | None | Lenvatinib | Yes | Yes | 2 - 25 |
| NCT04183062 | Bone Sarcoma | II | No | First line + Second Line | None | BIO-11006 | No | Yes | 5 - 21 |
| NCT04195555 | Solid Tumor | II | No | Second and other | IDH1 genetic alterations | Ivosidenib | Yes | No | 1 - 21 |
| NCT04238819 | Solid Tumor | I | No | Second and other | None | Abemaciclib | Yes | Yes | < 18 |
| NCT04294511 | OS | II | No | First line | None | Camrelizumab | Yes | Yes | 14 – 65 |
| NCT04308330 | Solid Tumor | I | No | Second and other | None | Vorinostat | Yes | Yes | 1 – 30 |
| NCT04320888 | Solid Tumor | II | No | Second and other | activating RET mutations | Selpercatinib | Yes | No | 1 - 21 |

| | | | | | | | | | |
|---|---|---|---|---|---|---|---|---|---|
| NCT04351308 | OS | II | Yes | First line | None | Camrelizumbab versus apatinib | Yes | Yes | ≥ 12 |
| NCT04417062 | OS | II | No | Second and other | None | Olaparib + Ceralasertib | Yes | No | 12 - 30 |
| NCT04433221 | ALL Sarcoma | I/II | No | Second and other | IHC positive staining for GD2 or PSMA or Her2 or CD276 | CAR-T | Yes | Yes | ≥ 6 months - ≤80 years |
| NCT04483778 | Solid Tumor | I | No | Second and other | None | B7H3-specific CAR-T versus bispecific B7H3xCD19 CAR-T | Yes | | < 26 |
| NCT04544995 | Solid Tumor | I | No | Second and other | BRCAness mutational signature (mutational signature 3) | Niraparib + Dostarlimab | Yes | No | 6 months – 17 years |
| NCT04595994 | ALL Sarcoma | I/II | Yes | Second and other | None | Selinexor | No | Yes | 18 - 80 |
